# Early and late neuropathological features of meningoencephalitis associated with Maraba virus infection

**DOI:** 10.1590/1414-431X20208604

**Published:** 2020-03-31

**Authors:** A. Maia-Farias, C.M. Lima, P.S.L. Freitas, D.G. Diniz, A.P.D. Rodrigues, J.A.S. Quaresma, C.W. Picanço Diniz, J.A. Diniz

**Affiliations:** 1 Laboratório de Microscopia Eletrônica, Instituto Evandro Chagas, Belém, PA, Brasil; 2 Laboratório de Neurodegeneração e Infecção, Instituto de Ciências Biológicas, Hospital Universitário João Barros Barreto, Universidade Federal do Pará, Belém, PA, Brasil; 3 Núcleo de Medicina Tropical, Universidade Federal do Pará, Belém, PA, Brasil

**Keywords:** Maraba virus, Vesiculovirus, Microglia, Astrocytes, Cytokines, Nitric oxide

## Abstract

Maraba virus is a member of the genus *Vesiculovirus* of the *Rhabdoviridae* family that was isolated in 1983 from sandflies captured in the municipality of Maraba, state of Pará, Amazônia, Brazil. Despite 30 years having passed since its isolation, little is known about the neuropathology induced by the Maraba virus. Accordingly, in this study the histopathological features, inflammatory glial changes, cytokine concentrations, and nitric oxide activity in the encephalon of adult mice subjected to Maraba virus nostril infection were evaluated. The results showed that 6 days after intranasal inoculation, severe neuropathological-associated disease signs appeared, including edema, necrosis and pyknosis of neurons, generalized congestion of encephalic vessels, and intra- and perivascular meningeal lymphocytic infiltrates in several brain regions. Immunolabeling of viral antigens was observed in almost all central nervous system (CNS) areas and this was associated with intense microglial activation and astrogliosis. Compared to control animals, infected mice showed significant increases in interleukin (IL)-6, tumor necrosis factor (TNF)-α, interferon (INF)-γ, MCP-1, nitric oxide, and encephalic cytokine levels. We suggest that an exacerbated inflammatory response in several regions of the CNS of adult BALB/c mice might be responsible for their deaths.

## Introduction

Maraba virus is a member of the genus *Vesiculovirus* and belongs to the family *Rhabdoviridae*, which was isolated from a group of 70 female phlebotomines captured on the main road of the Serra Norte region, Parauapebas county, state of Pará, Brazil. The genus *Vesiculovirus* comprises 16 species, including the Indiana, New Jersey, and Alagoas viruses that cause vesicular stomatitis in domestic animals (1), and the Piry, Chandipura, and Isfahan viruses that cause acute febrile illness, or in more severe cases, invade the central nervous system (CNS), producing meningoencephalitis in humans (2).

A neutralization test result suggested that a patient may have been exposed to Maraba virus (3). Infection of Vero cells showed that the pathogenesis of this virus is similar to that found in the Indiana and New Jersey *Vesiculovirus* (3). Our previous findings with this virus species in new-born nostril-infected mice showed necrosis and pyknosis of neurons in several regions of the CNS, including the cerebellum, hippocampus, and striatum, leading to death approximately 18 h after inoculation (4).

Some studies have shown that the initial inflammatory response to vesicular stomatitis virus (VSV) neuroinvasion is a consequence of reactive astrocytes and microglia activation (5). The glial response to viral infections promotes increased gene expression of complement factors, cytokines, nitric oxide, and neuropeptides that may be involved in both neuroprotection and neurodegeneration (6). CNS infection creates major difficulties for treatment due to the rapid onset of symptoms and disease progression with permanent or transient loss of memory, learning difficulties, and motor and sensorial deficits associated with neurodegeneration, which may end in dementia or death (7).

Currently, the genetically modified form - MG1 (mutations in the protein M L123W and G protein Q242R) of the Maraba virus has been used as a promising oncolytic agent, with higher efficiency than that of the VSV. In an *in vitro* study using human sarcoma tissues, MG1 infected more than 80% of the tested human sarcoma tissues, and subsequent *in vivo* treatment in rats led to a significant increase in long-lasting cures. In addition, this procedure induced the generation of an immune memory response that provided protection against subsequent tumor growth (8). However, after more than thirty years since the isolation of the Maraba virus, no study has been conducted to describe neuropathological changes triggered by this virus in adult animals. Thus, we investigated this question using a murine encephalitis model induced by Maraba virus to analyze the neuroimmune and histopathological responses triggered by this infection.

## Material and Methods

### Animals

The present study was approved by the Committee for Ethics in Research on the use of Animals of the Evandro Chagas Institute (IEC; protocol No. 06/2013/CEUA/IEC/SVS/MS). All procedures followed regulations established by the International Biosafety Committee for isolation procedures and techniques for the use of infectious agents belonging to biosafety level 3.

Inbred female albino adult mice of the BALB/c lineage (8 weeks old) and two-day-old neonates from the colony of the Evandro Chagas Institute Central Animal House were used. Animals were maintained in an animal house at the IEC Electronic Microscopy Laboratory in standard plastic cages (32×39×16 cm) with water and food *ad libitum* under controlled temperatures (23±2°C) and a 12-h light/dark cycle.

### Viral strain

Samples of the Maraba virus (BE AR 411459) were provided by the IEC Arbovirology and Hemorrhagic Fever Department.

### Virus titer

The viral titer of the samples was determined by plaque assays (9). Briefly, Vero cell monolayers in six-well plates were incubated with 100 μL serial (log 10) dilutions of the viral sample at 37°C for 1 h under gentle shaking every 15 min. After this incubation period, medium 199 (Sigma-Aldrich, USA) containing non-adsorbed virus was replaced with a semi-solid culture medium (3% carboxymethylcellulose in medium 199) supplemented with 2% fetal bovine serum, 100 U/mL penicillin, and 100 μg/mL streptomycin. After 7 days at 37°C, the cells were fixed and stained with 0.1% cresyl violet solution, 30% ethanol, and 20% formaldehyde in PBS, and the cell death zones (plaques) were counted. The viral titer was calculated by multiplying the number of plaques obtained from a given viral serial dilution and, subsequently, by the dilution factor, with the result being reported in plaque-forming units per milliliter (PFU/mL).

### Preparation of the viral inoculum

Brains of newborn mice infected with Maraba virus (0.2 g/animal) were macerated in phosphate-buffered saline (PBS) containing 100 U/mL penicillin and 100 mg/mL streptomycin. The infected brain homogenate was centrifuged at 6000 *g* for 15 min at 4°C. Adult mice had their nostrils instilled with 0.02 mL of the supernatant of the intranasally infected brain homogenate. Control animals received equal volumes of non-infective brain homogenates.

### Experimental groups

For the histopathological procedure, control (n=9) and infected (n=9) animals were perfused (as described below) on the second, fourth, and sixth days post-infection (dpi). For the animal samples used for immunohistochemistry, flow cytometry, ELISA, and Griess reagent experiments, the animals were divided into two euthanized groups either on the third and sixth day after inoculation.

### Perfusion and craniotomy

At three and six days after inoculation, when mice began to start and aggravate typical behavioral signs of preclinical disease, they were anesthetized with 2,2,2-tribromoethanol (Sigma-Aldrich, USA) (250 mg/kg body weight) and perfused transcardially with heparinized 0.9% saline solution (5000 IU/L), followed by 4% paraformaldehyde in 0.1 M phosphate buffer (pH 7.2–7.4). After perfusion, the brains were dissected, removed, and sectioned in the vibratome (Micron) on the horizontal plane (80-μm thick). Anatomical series of sections (1:5) were collected and subjected to immunohistochemical analysis, as described below.

### Histopathological processing

Sections were washed with 0.1 M PBS, pH 7.2–7.4, at room temperature, followed by progressive dehydration using an ethanol series (50, 70, 90, and 100%) followed by clarification in xylene (three immersions of 5 min each) at room temperature and immersion in two paraffin baths at 60°C. Subsequently, the samples were included in paraffin blocks and sectioned coronally (5-μm thick) using a rotating microtome (Leica, Switzerland). The sections were stained with hematoxylin and eosin, followed by optical microscopy (Axiophot, Zeiss, Germany) and digital imaging (AxioCam HRC, Zeiss).

### Immunohistochemistry for detection of the viral antigens GFAP and Iba1

Primary antibodies against anti-Maraba virus antigens, GFAP (Millipore^©^ #MAB360, Germany), and Iba1 (Wako^©^ #019-19741, USA) were used to selectively immunolabel viral antigens, astrocytes, and microglia, respectively. Brain sections from both infected and control animals were selected randomly and systematically, taking one in every five slices of 80 µm. The sections were washed in 0.1 M phosphate buffer, pH 7.2–7.4, and pre-treated for 60 min in 0.2 M boric acid solution, pH 9.0, at 70°C for antigenic recovery. After washing with PBS-5% Triton, the sections were transferred to a solution of 0.3% hydrogen peroxide in 0.1 M phosphate buffer, pH 7.2–7.4, for 15 min. After washing sections in PBS, the protocol for the Mouse-on-Mouse Kit (Vector Laboratories, USA) was followed using an anti-Maraba (1:100) polyclonal antibody obtained from ascitic mice, anti-GFAP, or anti-Iba1 primary antibodies at 1:400 in 0.1 M PBS, pH 7.2–7.4. Finally, the sections were washed in PBS and incubated in ABC (avidin-biotin complex, Vector Laboratories) for 30 min. The DAB-nickel glucose oxidase-diaminobenzidine protocol was used to reveal anti-Maraba, GFAP, and Iba-1 binding sites (10). Sections were mounted on gelatinized slides, left to dry at room temperature, and covered with Entellan (Merck, Germany) coverslips for further analysis.

### Cytokine analysis

Adult BALB/c mice (8 weeks old) were nasally instilled with either 10 μL saline or an equal volume of viral suspension and later euthanized at two time-points. The brains were then macerated in 2× PHEM buffer at a 1:5 ratio for cytokine and nitric oxide (NO) detection experiments.

### Immunoenzymatic assay (sandwich-type ELISA)

This technique was performed according to instructions from the kit manufacturer (BD OptEIATM, BD Biosciences, USA) to analyze the expression of interleukin (IL)-12p40 and transforming growth factor (TGF)-β1 cytokines. Ninety-six-well plates were incubated with capture antibody specific for each cytokine overnight at 4°C. Subsequently, the plates were washed with a solution containing 0.05% PBS and Tween 20 and then incubated with 200 μL of a blocking solution composed of PBS and 10% fetal bovine serum (FBS) for 1 h at room temperature. The plates were then washed again, followed by the addition of mouse brain suspension supernatants and serial dilutions of the standard curve, and incubated for 2 h at room temperature for cytokine TGF-β1 quantification. The samples were pre-treated with hydrochloric acid (HCl) for 1 h and then neutralized with sodium hydroxide (NaOH). At the end of this interval, the plates were washed five times and incubated with biotinylated detection antibody plus streptavidin bound to peroxidase for 1 h at room temperature. Plates were then washed and incubated with the chromogen (TMB, tetramethylbenzidine) for 15–30 min at room temperature and protected from light. After incubation, the reaction was quenched with 2 N sulfuric acid (H_2_SO_4_) and analyzed on an ELISA EL 800-BIO-TEK ELISA reader with a 450 nm filter.

### Flow cytometry

To analyze the expression of cytokines IL-6, TNF, interferon (IFN)-γ, IL-12p70, IL-10, and MCP-1, we followed the manufacturer's instructions for the BDTM-Cytometric Bead Array (CBA) Mouse Inflammatory kit (BD Biosciences).

Serial samples and serial dilutions of the standard curve were placed into properly identified tubes containing the cytokine detection capture antibodies mentioned above, incubated for 2 h, and then wash buffer was added to increase the sample volume and facilitate reading. The flow cytometer (BD FACSCanto II) was used to read the results, which were analyzed using FCAP Array 3.0 software (BD™ Cytometric Bead Array, USA).

### Griess reagent for nitrite quantification

We followed the manufacturer's instructions (Molecular Probes, USA) with some modifications. For nitrite quantification, formed by the spontaneous oxidation of NO under physiological conditions, we placed 50 μL of each supernatant from mouse brain suspensions in a 96-well plate together with 50 μL of the dilutions provided in the kit in appropriate buffer to produce the standard curve. Subsequently, 50 μL of Griess reagent, previously prepared with equal volumes of component A (N-(1-naphthyl) ethylenediamine dihydrochloride) and component B (sulfanilic acid), was incubated for 1 h at room temperature. The photometric reference sample was prepared by mixing 50 μL of the Griess reagent and 50 μL of deionized water. After incubation, the absorbance of the samples was measured using a spectrophotometer (Model EL 800, BIO-TEK, USA) with a 550 nm wavelength filter.

### Statistical analysis

Statistical analysis of the results of flow cytometry tests, ELISA, and Greiss reagent was performed using GraphPad Prism 5 software (USA) using the two-way ANOVA test and the Bonferroni multiple comparison post-test, with P<0.05.

## Results

### Clinical signs

Viral titer was 2.1×10^9^ PFU/mL and animals were inoculated with 42 PFU/mL. Although the infected animals showed viral antigens in the olfactory bulb at 3 dpi, no clinical signs associated with viral infection were detected. However, at 6 dpi, the infected animals showed strong clinical signs that included hunched postures, ruffled fur, and continuous circular motor ambulation. The death of all infected animals due to severe encephalitis occurred between the sixth and the seventh day after inoculation.

### Viral titration and histopathological analysis

**Figure 1:**
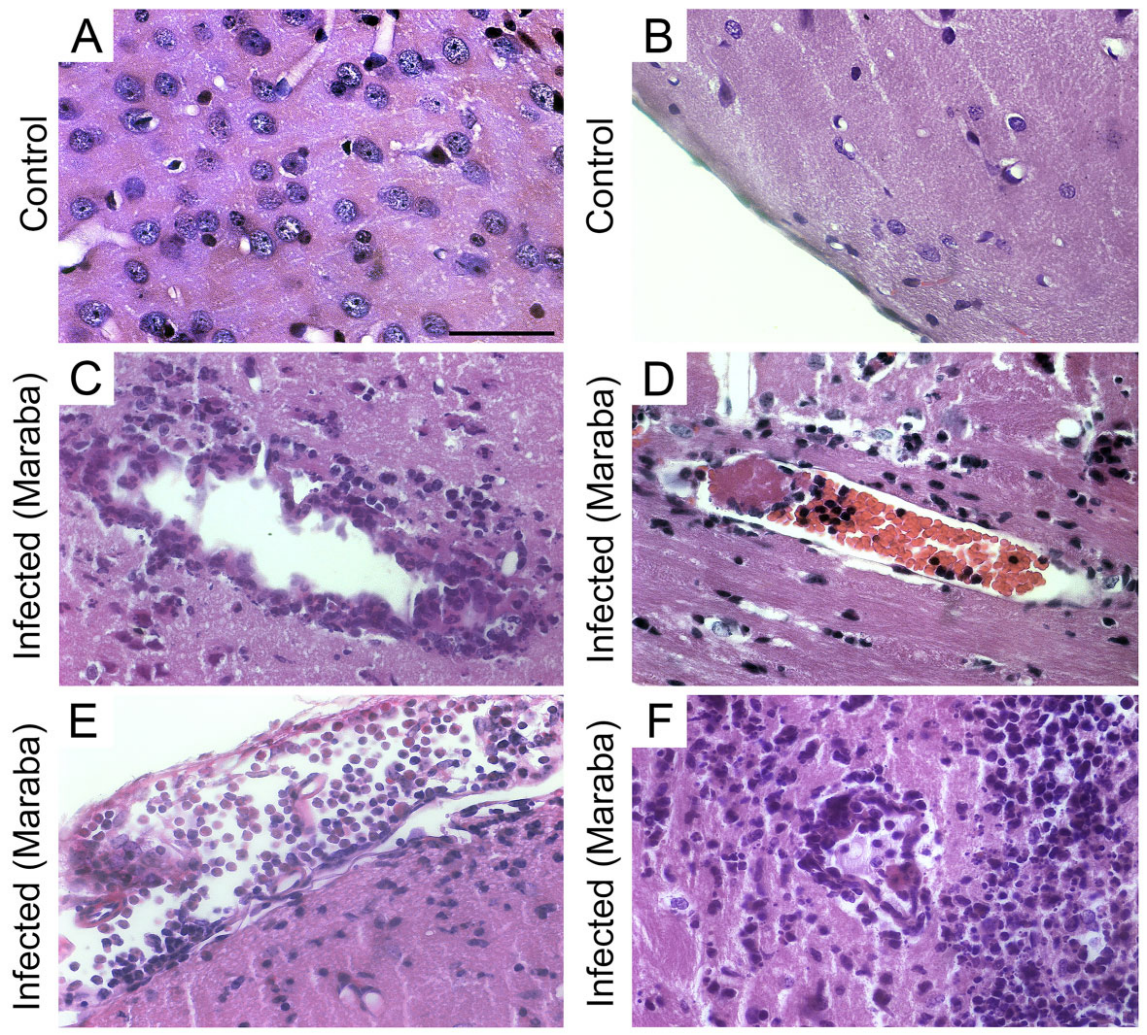
Photomicrographs of control (**A** and **B**) and infected mouse (**C** to **F**) sections, at 6 dpi (days post-infection), stained with hematoxylin/eosin. **A**, Hippocampal region and **B**, parietal cortex of control animal with normal pia mater; **C**, cortical area with perivascular inflammatory infiltrates with an extensive area of edema; **D**, cortical parenchyma; **E**, pia mater of the entorhinal cortex with inflammatory infiltrate; **F**, periventricular area with inflammatory infiltrate near the subependymal cell layer. Scale bar: 30 μm.

The viral titer was 2.1×10^9^ PFU/mL. Brains of the control animals showed no signs of inflammation (Figure 1A and B). At 2 dpi, the infected animals showed mild meningeal inflammatory lymphomononuclear infiltrate. The cerebral parenchyma showed severe congestion and discrete foci of lymphocytic infiltration, especially around vessels. These changes were mainly observed in the cerebral cortex and cerebellum. Neurons were generally preserved, with only a few discrete foci of pyknosis and neuronophagia. At 4 dpi, infected animals showed meningeal congestion, edema, lymphocytic inflammatory infiltrate, and rare neutrophils. The cerebral and cerebellar parenchyma showed areas of pyknosis and diffuse neuronophagy. There was congestion and proliferation of small vessels, with irregular endothelium and sometimes with hypertrophic cells. In addition, lymphocytic inflammatory infiltrate around the vessels, some neutrophils, and glial nodules were observed.

At 6 dpi, there was fragmented neural tissue with meningeal congestion and edema, permeated by inflammatory infiltrate of lymphocytes and rare neutrophils (Figure 1E). The cerebral and cerebellar parenchyma showed areas of pyknosis and neuronophagy distributed diffusely and associated with inflammatory infiltration of lymphocytes, plasma cells, and neutrophils of moderate intensity. Intense vascular congestion (Figure 1D), irregular endothelium sometimes with hypertrophic cells, and inflammatory infiltration of lymphocytes with neutrophils around vessels were also observed (Figure 1C). These infiltrates filled the spaces of Virchow-Robin. In the cerebral parenchyma, there were also frequent glial nodules and intense edema. Figure 1F shows intense infiltration of inflammatory cells in the parenchyma, blood vessels, and lateral ventricle wall.

### Distribution of viral antigens in the brain and immunostaining of astrocytes and microglia

**Figure 2:**
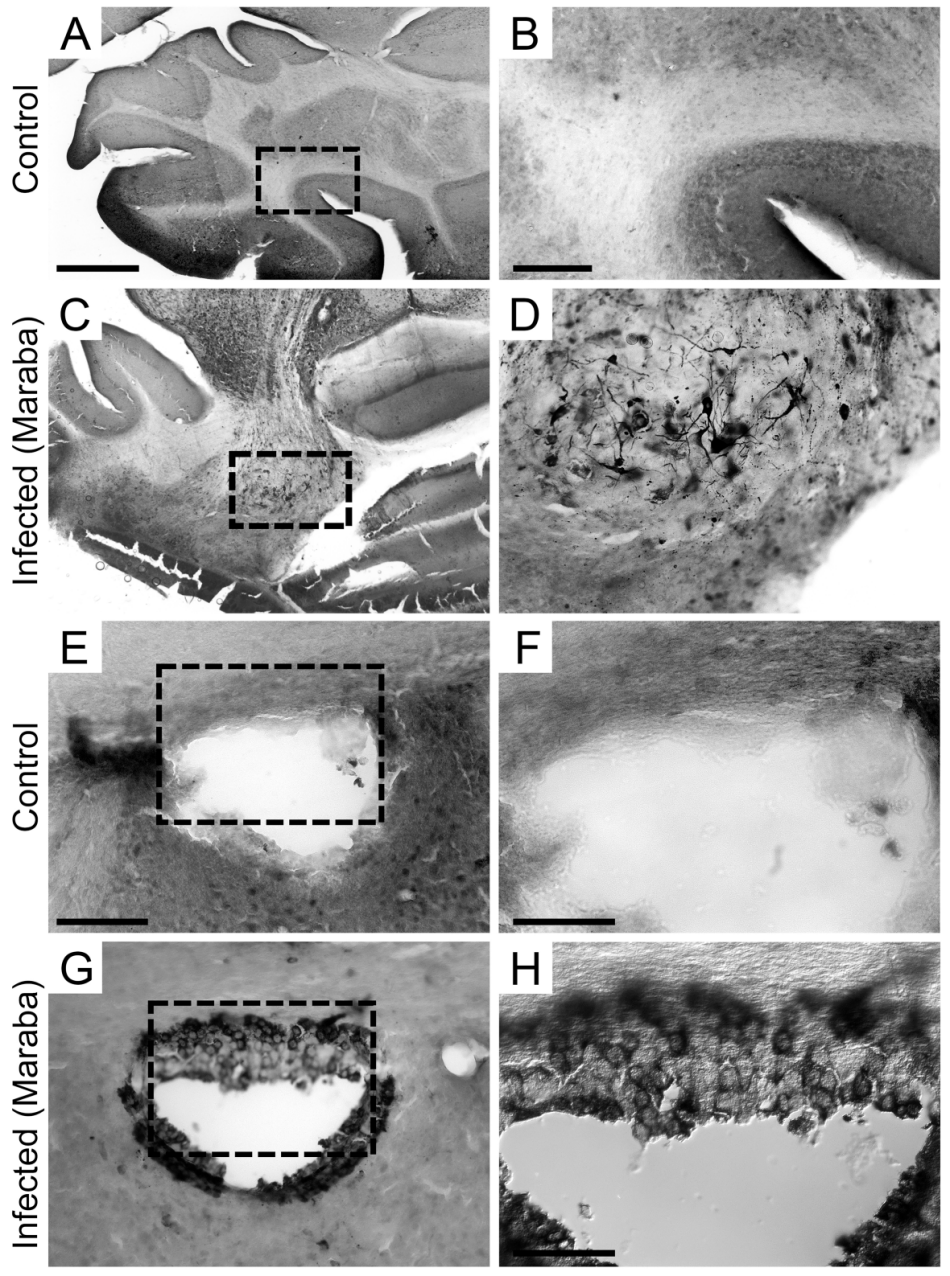
Photomicrographs of mouse brain sections immunolabelled for viral antigens. Control (**A**, **B**, **E**, **F**) and infected animals at 6 dpi (days post-infection) (**C**, **D**, **G**, **H**). Cerebellum from control (**A** and **B**) and infected mice (**C** and **D**); third ventricle wall from control (**E** and **F**) and infected mice (**G** and **H**). Dotted areas indicate areas of high-power images. Scale bars **A**, **C**: 650 μm; **B**, **D**: 150 μm; **E**, **G**: 50 μm; **F**, **H**: 30 μm.

**Figure 3:**
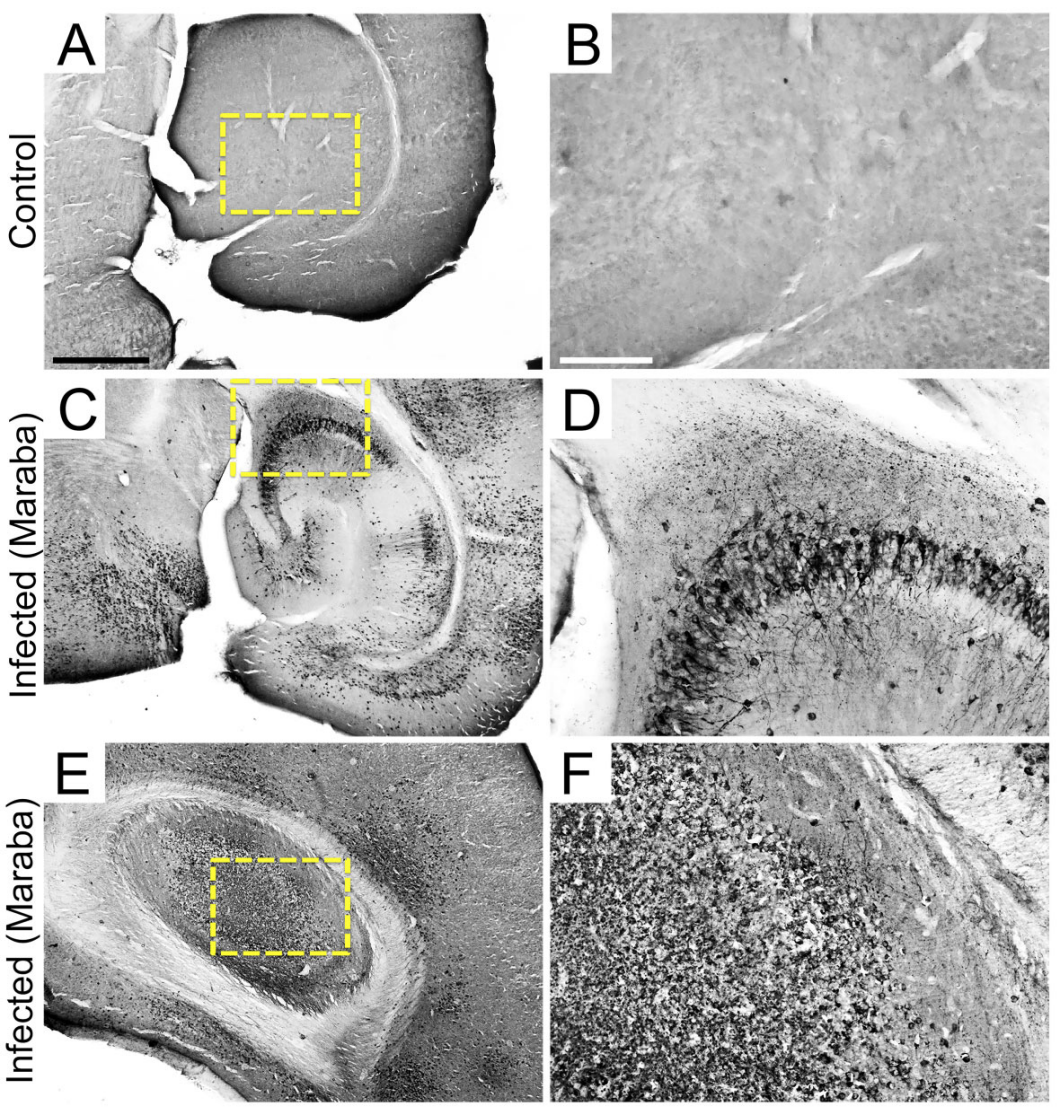
Photomicrographs after Maraba virus antigen immunolabelling of Control (**A**, **B**) and 6 days post-infection infected mouse sections (**C** to **F**). Yellow-dotted square areas indicate the regions of high-power images. Scale bars **A**, **C**, **E**: 650 μm; **B**, **D**, **F**: 150 μm.

Figures 2 and 3 illustrate control mice photomicrographs of immunolabelled sections for virus antigens taken from the cerebellum (Figure 2A and B), third ventricle wall (Figure 2E and F), and hippocampal formation (Figure 3A and B). As expected, control animals did not show viral antigens. On the third day after intranasal inoculation, viral antigens were detected in the olfactory bulb, olfactory pathways. In addition, small globular immunolabelling in the brainstem was also observed at 3 dpi, suggesting the presence of the virus within intracellular vesicles distant from the primary infection site (data not shown).

Brain sections from animals at 6 dpi showed viral antigens in several brain regions, including the olfactory bulb, frontal cortex, parietal, temporal, and occipital cortex, olfactory lobe, choroid plexus, septal area, and third ventricle wall (Figure 2G and H), 3rd and 4th ventricles, CA1, CA2, CA3 (Figure 3C and D), and ventral hippocampus (Figure 3E and 3F), internal capsule, dentate gyrus, basal ganglia, thalamus, cingulum, callosal fibers, brainstem, cerebellum (Figure 2C and D), and brain meninges.

**Figure 4:**
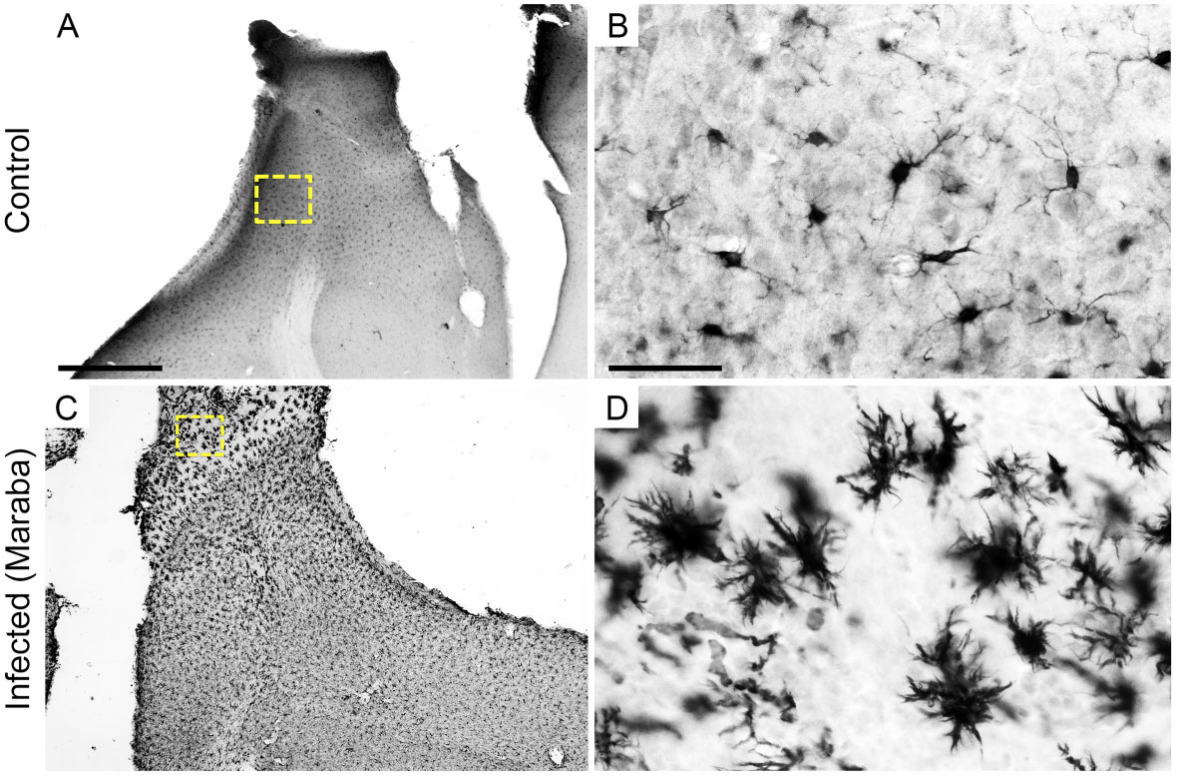
Photomicrographs of mouse brain sections immunolabelled for Iba-1. Microglia in the olfactory bulb of control (**A** and **B**) and infected mice (**C** and **D**) sections at 3 days post-infection immunolabelled for Iba-1. Yellow-dotted squared areas indicate the regions of high-power images. Scale bars **A** and **C**: 650 μm; **B** and **D**: 30 μm.

Activated microglia were observed on the olfactory bulb (Figure 4C and D) and olfactory pathways at 3 dpi. As expected, control animals did not show microglial activation. At 6 dpi, microglial activation coexisted with viral antigens in the same encephalic regions. In addition, amoeboid Iba-1-positive cells were observed at 6 dpi in infected mice, mainly in the cortical layers near the pia mater.

**Figure 5:**
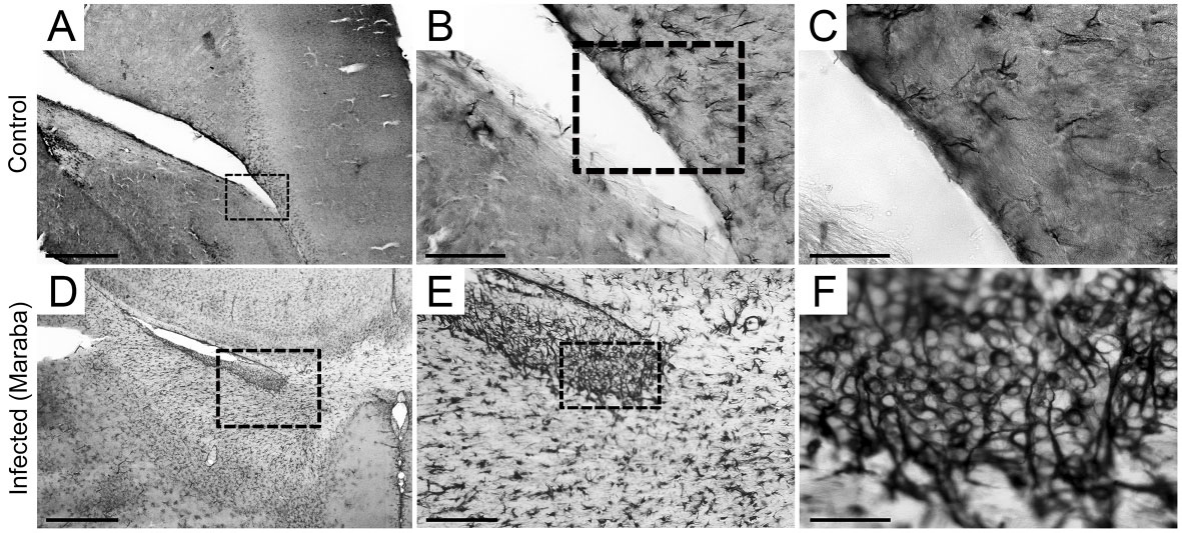
Low- and high-power photomicrographs of brain (lateral ventricle adjacent region) GFAP-immunolabelled sections of control (**A** to **C**) and infected mice at 6 days post-infection (**D** to **F**). The dotted areas indicate the regions of high-power images. Scale bars **A**, **D**: 650 μm; **B**, **D**: 50 μm; **C**, **F**: 30 μm.

**Figure 6:**
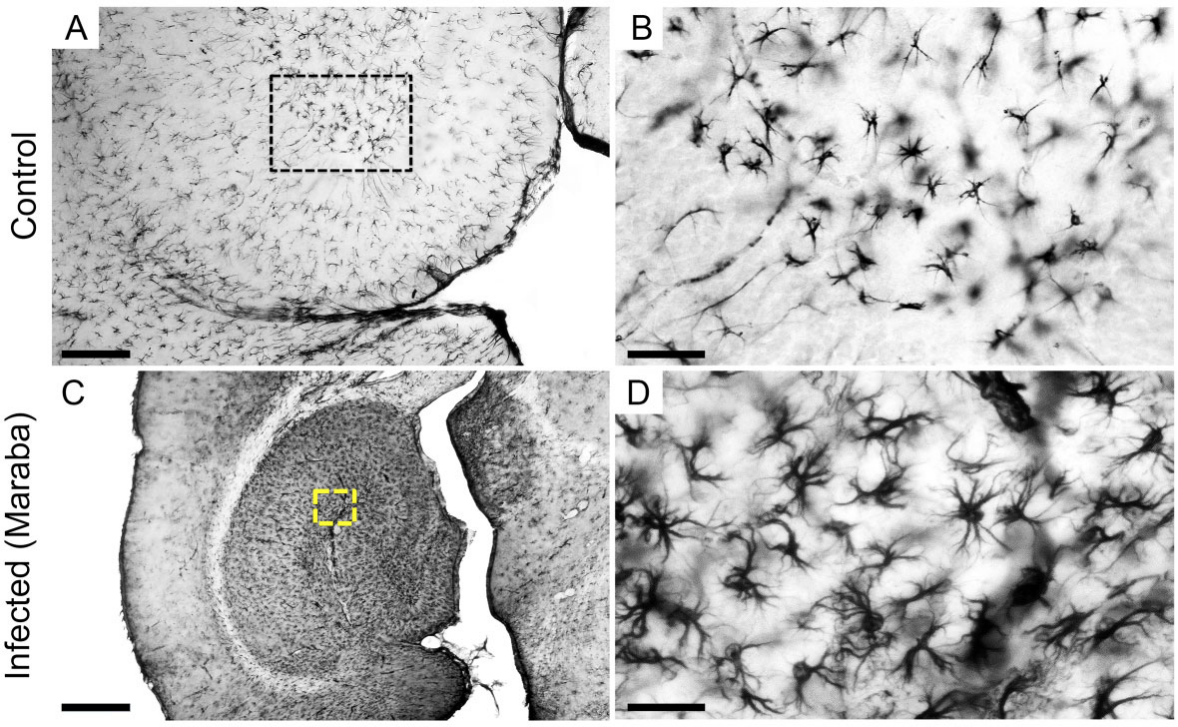
Low- and high-power photomicrographs of GFAP immunolabelled sections of control (**A** and **B**) and infected mice (**C** and **D**) at 6 days post-infection. The dotted areas indicate the regions of high-power images. Scale bars **A**, **C**: 650 μm; **B**, **D**: 30 μm.

GFAP immunohistochemistry did not show reactive astrocytes in the control group (Figure 5A–C and Figure 6A and B). Infected mouse brains at 3 dpi showed reactive astrocytes only in the olfactory bulb (data not shown), which spread subsequently at 6 dpi to the olfactory and frontal cortices, walls of the lateral ventricle (Figure 5D and F), and to third and fourth ventricles, septal region, fimbria, and hippocampus (Figure 6C and D).

### Quantification of cytokines and NO

**Figure 7:**
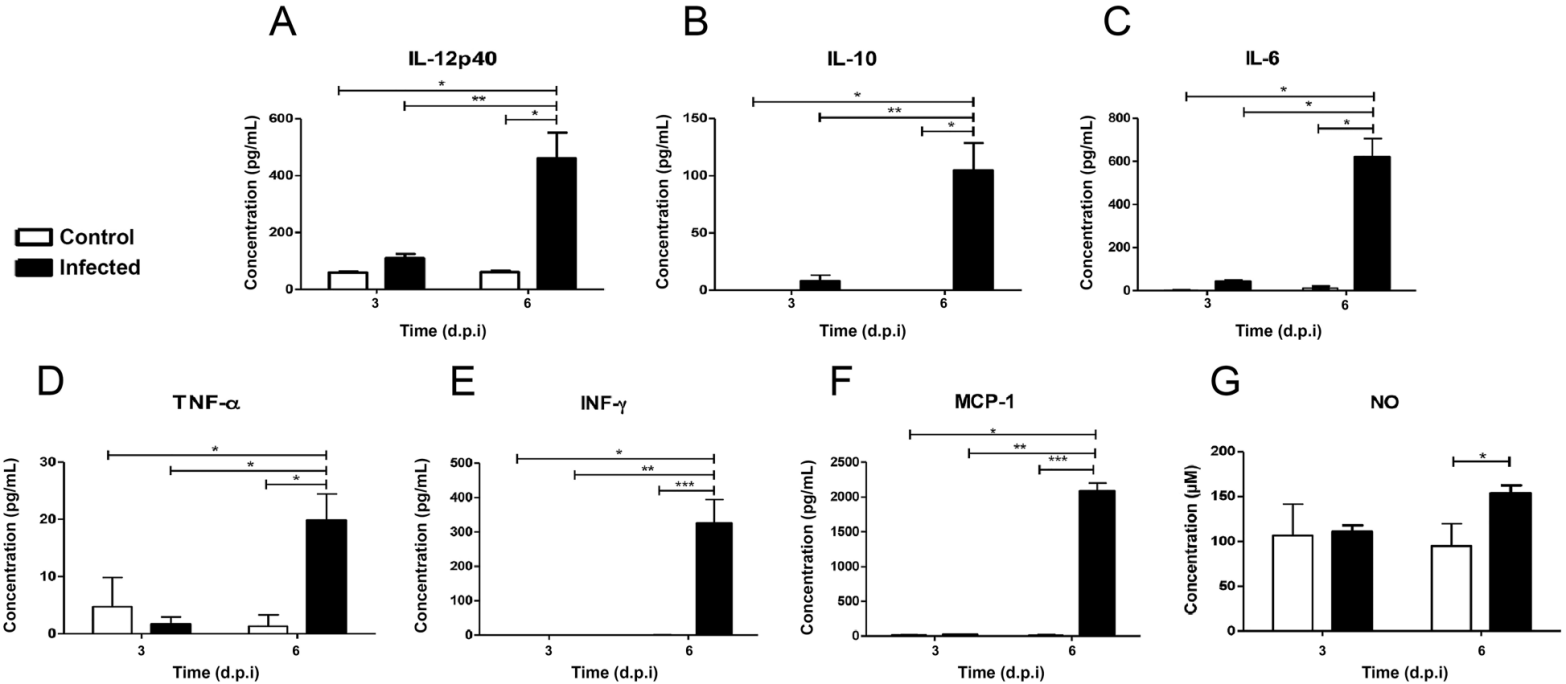
Expression of IL-12p40, IL-10, IL-6, TNF-α, INF-γ, MCP-1, and NO in the supernatants of control and infected brain homogenates of mice at 3 and 6 d.p.i. (days post-infection). Data are reported as mean±SD. *P<0.001; **P<0.01; ***P<0.05 (two-way ANOVA and Bonferroni post-test for multiple comparisons).

Analysis of the expression of TGF-β and IL12-p70 cytokines did not show significant differences between the control and infected mice at the assessed time points (data not shown). However, compared to the other groups at 6 dpi, a significant increase in the expression of IL-12p40, IL-10, TNF-α, IL-6, INF-γ, and monocyte chemotactic protein-1 (MCP-1) was observed. NO expression in infected animals was significantly higher at 6 dpi than that in the control animals during the corresponding time (Figure 7).

## Discussion

In the present research, we described histopathological changes, viral dissemination, glial reaction, and expression of TNFα, TGF-β, INF-γ, IL-6, IL-10, IL-12p40, IL-12p70, MCP-1, and NO in the brains of young adult mice after Maraba virus-induced meningoencephalitis.

Our histopathological findings showed an intense and fatal inflammatory process including pyknosis and necrosis, edema, vascular congestion, and lymphocytic infiltrates in vessels and meninges leading to death between the sixth and eighth day after inoculation. Most viral infections in the CNS are transient but may result in disability or death. Several RNA and DNA viruses enter the CNS through axonal transport in the peripheral nervous system (11) and may induce nerve tissue injury by direct replication and lysis of host cells, and the activation of innate and adaptive immune responses is associated with apoptosis or autophagy (11). Viruses may invade the CNS through the hematogenic pathway due to changes in the endothelial cells of blood vessels through the blood-brain barrier or through the interior of infected cells that move through the endothelium (12,13). Viruses can also be transported axonally, as observed in a murine VSV encephalitis model in which primary infection is in the olfactory receptor neurons, followed by dissemination to the olfactory bulb and other CNS areas (14).

Nostril inoculation of the *Vesiculovirus* New Jersey serotype in rats showed viral antigens caudally disseminated from the olfactory bulb, ventricular system, and central canal of the spinal cord (15). Our results suggested that Maraba virus was both axonally and hematogenously disseminated in CNS areas. Indeed, Maraba virus antigens seemed to be transported from the olfactory bulb towards olfactory-adjacent areas, and they appeared on distant, unrelated CNS areas.

Here, it was shown earlier in the disease that microglial and astrocytic activation were restricted to the olfactory bulb, whereas later, after viral antigen dissemination, microglial activation and reactive astrocytosis were present in all regions where viral antigens were detected. Bi and Reiss (16) showed that VSV-infected (via nostril) adult BALB/c mice had astrogliosis in the olfactory bulb at 1 dpi and that microglial activation was present at 3 dpi in the olfactory bulb. Machado et al. (17) also observed intense labelling of reactive astrocytes in the olfactory bulb and hippocampus of adult C57BI6 mice after 6 days of infection with VSV. In addition, Chauhan et al. (18) showed that the infection of astrocytes and microglia by VSV *in vivo* induces the production of inflammatory mediators. The intensity of the glial reaction seems to be directly proportional to the production of a variety of growth factors, cytokines, NO, and neuropeptides, which, in large quantities, are associated with the development of neurodegeneration (19,5).

Our findings showed significant increases in the levels of IL-6, TNF-α, INF-γ, MCP-1, and NO late in disease progression, which may have contributed to cell death and lymphocytic infiltration at the end of infection. The cytokine TNF-α, for example, can induce microglial activation (20) and proliferation of astrocytes (21), followed by the expression of other cytokines, necrosis, and cellular apoptosis (22,23). Consistently, in the Japanese encephalitis virus model, TNF-α is associated with the death of neurons, as well as neutralizing antibodies involved in the reduction of virus-induced neuronal death (24). TNF-α stimulates the production of IL-6, which together with other cytokines, IFN-γ for example, can induce reactive gliosis, local synthesis of neurotrophic factors, or even neural degeneration (25). IL-6 is a pleiotropic cytokine acting in the CNS under homeostatic, inflammatory, and disease conditions, and performing dual actions (26). Many cells in the CNS produce IL-6 (27), but astrocytes are the main producers (26,28). Indeed, the results of experimental viral infections *in vitro* and *in vivo* confirm that the main sources of IL-6 in the CNS are astrocytes (29).

Our study showed that on the sixth dpi there was an intense increase in MCP-1 levels, which may be directly related to the presence of inflammatory infiltrates in the brain parenchyma, in the meninges and around the vessels. In agreement, *in vitro* studies show that MCP-1 induces potent chemotaxis of macrophages, CD4 and CD8 activated lymphocytes (30), and effective inflammatory infiltration in inflammatory disease models (31). In addition, MCP-1 may contribute to increased INF-γ levels because MCP-1 induces the development of the Th1 response, with consequent increases in INF-γ expression by these cells (32). In addition, INF-γ may contribute to microglial activation, upregulate microglial MHC-II expression, increase phagocytosis and cytokine production (33), and induce increased production of microglial NO (22). Sun et al. (34) have shown that IFN-γ strongly promotes IL-6 production *in vivo* and significantly reduces the levels of IL-6 after the use of anti-IFN-γ antibodies. IFN-γ, which is produced primarily by activated T cells and NK cells, plays an important role in immune defense and may exert neuroprotection during viral infections (35). However, prolonged expression of IFN-γ in the CNS can also produce damage in neurons and glial cells (36). It seems that acute increases in IFN-γ levels prevent fatal encephalitis in cases of viral infection (37); however, in our study, increases in these cytokines were not sufficient to ameliorate the infectious process, encephalic damage, and reduced pro-inflammatory status.

Although this work showed an increase in IL-12p40 homodimer expression, a decrease in the bioactive IL-12p70 heterodimer was detected, probably due to the low IL-12p35 synthesis. Because we did not quantify this synthesis in the present work, this cause-effect relationship remains to be resolved. However, it is reasonable to suggest that the decrease of the bioactive IL-12p70 heterodimer cannot explain the progression of the inflammatory and histopathological processes generated by meningoencephalitis. In addition, our work showed that there was a significant increase in the expression of the anti-inflammatory cytokine IL-10, which occurred on the last day of the infection and perhaps because of this fact, it was not effective in reducing the inflammatory response. Indeed, IL-10 can prevent inflammation of the nervous system, but its effectiveness depends on the timing of its production (38).

Taken together, our results suggested that the CNS dissemination of Maraba virus antigens induced an exacerbated inflammatory response characterized by intense reactive astrocytosis and microglial activation and a significant increase in the levels of pro-inflammatory cytokines TNFα, INF-γ, IL-6, MCP-1, and NO, and these events led to intense clinical signs followed by death of all infected animals.
